# Adiponectin triggers breast cancer cell death via fatty acid metabolic reprogramming

**DOI:** 10.1186/s13046-021-02223-y

**Published:** 2022-01-05

**Authors:** Duc-Vinh Pham, Pil-Hoon Park

**Affiliations:** 1 College of Pharmacy, Yeungnam University, Gyeongsan, Korea; 2 Research Institute of cell culture, Yeungnam University, Gyeongsan, Korea

**Keywords:** Adiponectin, Breast cancer, Cancer metabolism, Lipophagy, SIRT-1, SREBP-1

## Abstract

**Background:**

Adiponectin, the most abundant adipokine derived from adipose tissue, exhibits a potent suppressive effect on the growth of breast cancer cells; however, the underlying molecular mechanisms for this effect are not completely understood. Fatty acid metabolic reprogramming has recently been recognized as a crucial driver of cancer progression. Adiponectin demonstrates a wide range of metabolic activities for the modulation of lipid metabolism under physiological conditions. However, the biological actions of adiponectin in cancer-specific lipid metabolism and its role in the regulation of cancer cell growth remain elusive.

**Methods:**

The effects of adiponectin on fatty acid metabolism were evaluated by measuring the cellular neutral lipid pool, free fatty acid level, and fatty acid oxidation (FAO). Colocalization between fluorescent-labeled lipid droplets and LC3/lysosomes was employed to detect lipophagy activation. Cell viability and apoptosis were examined by MTS assay, caspase-3/7 activity measurement, TUNEL assay, and Annexin V binding assay. Gene expression was determined by real time-quantitative polymerase chain reaction (RT-qPCR) and western blot analysis. The transcriptional activity of SREBP-1 was examined by a specific dsDNA binding assay. The modulatory roles of SIRT-1 and adiponectin-activated mediators were confirmed by gene silencing and/or using their pharmacological inhibitors. Observations from in vitro assays were further validated in an MDA-MB-231 orthotopic breast tumor model.

**Results:**

Globular adiponectin (gAcrp) prominently decreased the cellular lipid pool in different breast cancer cells. The cellular lipid deficiency promoted apoptosis by causing disruption of lipid rafts and blocking raft-associated signal transduction. Mechanistically, dysregulated cellular lipid homeostasis by adiponectin was induced by two concerted actions: 1) suppression of fatty acid synthesis (FAS) through downregulation of SREBP-1 and FAS-related enzymes, and 2) stimulation of lipophagy-mediated lipolysis and FAO. Notably, SIRT-1 induction critically contributed to the adiponectin-induced metabolic alterations. Finally, fatty acid metabolic remodeling by adiponectin and the key role of SIRT-1 were confirmed in nude mice bearing breast tumor xenografts.

**Conclusion:**

This study elucidates the multifaceted role of adiponectin in tumor fatty acid metabolic reprogramming and provides evidence for the connection between its metabolic actions and suppression of breast cancer.

**Supplementary Information:**

The online version contains supplementary material available at 10.1186/s13046-021-02223-y.

## Background

A large body of evidence indicates that obesity is closely associated with an increased incidence of various cancers [1]. One plausible mechanism underlying the adiposity-cancer connection is dysregulated production of adipokines, a group of hormones produced from adipose tissues, in obese individuals [2]. Of the various adipokines, adiponectin has shown potent anti-cancer effects via multiple mechanisms, and, interestingly, its levels are downregulated during obesity. Therefore, impaired adiponectin signaling has been proposed to contribute to obesity-associated cancer development and progression [3]. Until now, most efforts in elucidating the role of adiponectin in deciding the fate of the cancer cells have been made in the context of breast cancer, where a causal relationship between obesity and carcinogenesis has been well established. In particular, recent studies have documented that adiponectin induces breast cancer cell death and growth arrest by regulating a wide range of cellular events, such as autophagy, tumor-promoting inflammation, inflammasomes activation, and oncogenic signals driven by leptin and estrogen [4–8]. Nevertheless, the detailed molecular mechanisms by which adiponectin modulates breast cancer cell death and survival still need to be further clarified.

Tumor metabolic reprogramming is an important adaptive mechanism that provides cancer cells with metabolic plasticity to boost cell survival and infinite proliferation in a challenging tumor microenvironment [9]. Alterations in cellular metabolism, such as increased glutaminolysis and aerobic glycolysis have long been described as common metabolic phenotypes in cancer cells. In addition, recent advances in the investigation of cancer metabolism have led to the discovery of new metabolic phenotypes that control tumor growth in a context-dependent manner [10]. Although tumor-specific lipid metabolic remodeling has only recently received much attention, compelling evidence has demonstrated its indispensable roles in different aspects of tumorigenesis [11]. Many cancer cell types show increased dependence on *de novo* lipogenesis, despite the abundance of exogenous lipids in tumor microenvironment. For example, genes involved in fatty acid synthesis (FAS) pathway, including sterol regulatory element-binding protein 1 (SREBP-1), fatty acid synthase (FASN), acetyl-coA carboxylase 1 (ACC1), and ATP citrate lyase (ACLY), have been reported to be overexpressed in breast tumor tissues and are essential for the survival of breast cancer cells [12–15]. A unique feature of cancer lipid metabolism is the coexistence of lipogenesis and lipolysis, in which *de novo* lipogenesis may be accompanied by enhanced fatty acid oxidation [16, 17]. Together, the altered lipid metabolism supports cancer cells through the maintenance of energy homeostasis and other lipid-dependent cellular processes, such as membrane biogenesis, protein modification, and signal transduction [18].

Having been known as an adipokine with a wide spectrum of cellular metabolic activities, it is surprising that the impact of adiponectin on cancer metabolism is under-investigated. Under physiological conditions, adiponectin acts as a negative modulator of lipid biosynthesis. For example, it reduces the expression of SREBP-1 in hepatocytes, which leads to the downregulation of enzymes in the FAS pathways [19]. Likewise, adiponectin induces phosphorylation and inactivation of ACC-1, an important rate-limiting enzyme of lipogenesis, through activation of AMPK in skeletal muscle cells [20]. Although direct action of adiponectin on cancer cell-specific lipid metabolism has not uncovered yet, adiponectin-modulated signaling pathways have been implicated in the control of both tumor growth and cellular lipid metabolism. Indeed, many of the anti-cancer activities of adiponectin have been referred to AMPK activation, a master regulator of cellular metabolism [21, 22]. Similarly, suppression of mTOR, a central player in maintenance of lipid homeostasis [23], crucially contribute to the modulation of tumor growth by adiponectin [24]. In addition, as autophagy is an essential mechanism for the degradation of intracellular lipids [25], activation of autophagy by adiponectin previously observed in cancer cells is also expected to exert some effects on lipid metabolism [4].

Sirtuin 1 (SIRT1), a member of nicotinamide adenine dinucleotide (NAD+)-dependent class III histone deacetylases, plays a crucial role in regulation of cellular metabolism by directly controlling the function of numerous transcription factors through its deacetylase activity [26]. Although it has received much attention, the role of SIRT-1 in cancer growth is still controversial. Several studies have found that SIRT-1 is upregulated in breast tumor tissues and acts as an oncogene [27, 28]. On the contrary, decreased expression of SIRT-1 has been reported in human invasive breast tumors [29]. In addition, overactivation of SIRT-1 led to a reduction in breast tumorigenesis, estrogen receptor (ER)-dependent cell proliferation, and metastasis [30]. Surprisingly, while the evidence clearly indicates a context-dependent role of SIRT-1 in breast cancer, little is known about the involvement of cellular metabolic modulation by SIRT-1 in its pro-oncogenic and anti-oncogenic actions. Moreover, a previous study showed that adiponectin enhances the expression of SIRT-1 via induction of reactive oxygen species (ROS) generation [31], raising a possibility of the implication of SIRT-1 in the anti-breast cancer effects of adiponectin.

In the present study, we found that globular adiponectin causes extensive changes in fatty acid metabolism in breast cancer cells. To do so, adiponectin suppressed FAS through blockage of SREBP-1-mediated transcription of FAS genes. In addition, it increases lipolysis by activating lipophagy and promotes fatty acid oxidation. Together, these changes trigger an imbalance in cellular lipid homeostasis, which in turn leads to the disruption of lipid raft structure and lipid raft-dependent signal transduction. We have also demonstrated that SIRT-1 activation is essentially required for fatty acid metabolic reprogramming and apoptosis induction by adiponectin in breast cancer cells.

## Methods

### Cell culture and reagents

MDA-MB-231, MCF-7, and T47D breast cancer cell lines were obtained from American Type Culture Collection (Rockville, MD, USA) and routinely cultured in RPMI 1640 media (MDA-MB-231 cells) or DMEM high Glucose (MCF-7 and T47D cells) supplemented with 10% FBS and antibiotics (1% penicillin/streptomycin). MDA-MB-231 cells stably expressing firefly luciferase (MDA-MB-231-luc) were kindly provided by Dr. Sang-Gyun Kim in Daegu Gyeongbuk Medical Innovation Foundation (DGMIF). Cells were maintained at 37 ^o^C in an incubator under a humidified atmosphere of 5% CO_2_. All reagents for cell culture were procured from HyClone Laboratories (South Logan, UT, USA).

Recombinant human globular adiponectin (gAcrp; #450-21) was purchased from PeproTech (Rocky Hill, NJ, USA). Recombinant Human Wnt-3a Protein (#5036-WN) was acquired from R&D Systems (Minneapolis, MN, USA). TVB-3166 (#SML1694), palmitic acid (#P5585), and MHY1485 (#SML0810) were purchased from Sigma-Aldrich (St. Louis, MO, USA). EX527 (#2780) and dorsomorphin dihydrochloride (compound C; #3093) were purchased from Tocris Bioscience (Bristol, UK). Rapamycin (#9904) was procured from Cell Signaling Technology Inc. (Beverly, MA, USA). Antibodies against p-AMPKα (#2531), AMPKα (#2532), non-phospho (active) β-catenin (#8814), FASN (#3180), p-Akt (# 9018), Akt (#9272), LC3 (#2775), p-mTOR (#2971), mTOR (# 2972), Bax (#5023), Bcl2 (#3498), Acetylated lysine (#9441), p62 (#5114), and Beclin-1 (#3738) were purchased from Cell Signaling Technology Inc.; antibodies for LRP-6 (#sc-25317), SIRT-1 (#sc-15404), and ubiquitin (#sc-8017) were purchased from Santa Cruz Biotechnology (Dallas, TX, USA); antibodies for Atg5 (#PA1-46178) and β-actin (#MA5-15739) were purchased from Thermo Scientific (Waltham, MA, USA); antibodies for SREBP-1 (#ab191857) and p-LRP6 (#GTX62033) were purchased from Abcam (Cambridge, MA, USA) and GeneTex Inc. (Irvine, CA, USA), respectively. HRP-conjugated secondary antibodies against rabbit (#31460) and mouse (#31430) IgG were procured from Thermo Scientific. The biotinylated anti-rabbit secondary antibody (#BA-1000) was obtained from Vector Laboratories Inc. (Burlingame, CA, USA).

### Cell viability assay

Cells were seeded in 96-well plates at a density of 2 × 10^4^ cells/well. After the indicated treatments, cells in each well were incubated with 20 μL of MTS reagent (#G3580; Promega Corporation, Madison, WI, USA) for 1-3 h at 37 ^o^C. Cell viability was determined by measuring the absorbance of the resultant formazan dye at 490 nm using a SPECTROstar Nano microplate reader (BMG Labtech Inc., Ortenberg, Germany).

### Caspase-3/7 activity assay

The caspase-3/7 activity was measured using Caspase-Glo® 3/7 Assay System (#G8091; Promega Corporation) as previously described [7]. Briefly, breast cancer cells were plated in white-walled 96-well plates at an initial density of 2 × 10^4^ cells/well for 16 h, followed by treatments as indicated. Finally, the cells were incubated with a luminogenic substrate Ac-DEVD-pNA for 1 h, and the luminescence produced by the cleavage of Ac-DEVD-pNA was recorded using a Spark™ 10M multimode microplate reader (Tecan, Mannedorf, Switzerland).

### Annexin V assay

Apoptotic cells were detected by FITC Annexin V Apoptosis Detection Kit (#640922; BioLegend, San Diego, CA, USA) in compliance with the manufacturer’s instructions. After trypsinization, the cells were collected and properly washed with washing buffer. Cells were then incubated with a staining solution containing FITC-Annexin V (1 μg/mL) and 7-AAD (2.5 μg/mL) for 15 min at room temperature. Finally, the percentage of apoptotic cells was determined by flow cytometry analysis using BD FACSCalibur™ (BD Biosciences, San Jose, CA, USA).

### TUNEL assay

TUNNEL assay was carried out to detect late apoptotic cell population using the TUNEL Assay Kit - FITC (#ab66108; Abcam) according to the manufacturer’s guidelines. Briefly, 10^6^ cells were fixed with 1% paraformaldehyde for 15 min on ice, followed by permeabilization with ice-cold 70% ethanol for 30 min. Fixed cells were then incubated with a staining solution containing TdT enzyme, FITC-dUTP, and reaction buffer for 60 min at 37 ^o^C. After washing 2 times with rinse buffer, the cells were subjected to flow cytometry analysis to determine the percentage of FITC-positive cells.

### Determination of cellular free fatty acids (FFA)

Cellular long-chain free FFA levels were measured using Free Fatty Acid Assay Kit from Abcam (#ab65341). 10^6^ cells were homogenized in 200 μL of 1% Triton X-100 in chloroform. The organic phase was subsequently separated by centrifugation, followed by air-drying at 50 ^o^C for 60 min. The dried lipids were dissolved in Fatty Acid Assay Buffer and converted into acyl-CoA by incubating with 2 μL of Acyl-CoA Synthetase (ACS) Reagent for 30 min at 37 ^o^C in a black 96-well plate. Then, 50 μL of reaction buffer was added into each well to oxidize acyl-CoA. The fluorescence of the resultant product was measured using Spark™ 10M multimode microplate reader at Ex/Em= 535/587 nm.

### Measurement of fatty acid oxidation (FAO)

FAO in breast cancer cells was monitored using the Fatty Acid Oxidation Complete Assay Kit from Abcam (#ab222944) according to a protocol described previously [32]. In brief, cells were seeded in a 96-well black plate with a clear bottom at a density of 2 × 10^4^ cells/well. After treatment with gAcrp as indicated, cells were washed two times with FA-Free Measurement Media (containing 0.5 mM L-carnitine and 2.5 mM glucose in base measurement media), followed by adding 90 μL of FA measurement media (containing 150 μM oleate-BSA conjugate) and 10 μL of extracellular O2 consumption reagent. After sealing each well with 100 μL of high sensitivity mineral oil, the fluorescence was read every 1.5 min for 60 min at Ex/Em of 360/670 using Spark™ 10M multimode microplate reader.

### Measurement of cellular neutral lipids

For determination of intracellular neutral lipid levels, cells were labeled with Bodipy 493/503 (#D3922, Thermo Scientific) as previously described [32]. Neutral lipid accumulation was represented by the uptake of Bodipy 493/503 uptake measured by flow cytometry.

For observation of intracellular lipid accumulation, cells in 8-well glass slides were incubated with Bodipy 493/503 (2 μM) or Nile red (300 nM) (#72485, Sigma Aldrich) for 15 min at 37 ^o^C to label lipid droplets. The fluorescent-labeled lipid droplets were then observed under a confocal microscopy (Nikon, Tokyo, Japan).

### SREBP-1 DNA binding activity assay

The specific transcription factor DNA binding activity was examined using the SREBP-1 Transcription Factor Assay Kit from Abcam (#ab133125) according to the manufacturer’s protocol. First, nuclear extracts were prepared from 10^7^ cells using the Nuclear Extraction Kit (ab113474; Abcam). Then, 20 μg of nuclear extract in Transcription Factor Binding Buffer was added into a 96-well plate to immobilize SREBP-1 on the well surface by binding with a pre-designed specific double-stranded DNA (dsDNA) sequence. After overnight incubation at 4 ^o^C, dsDNA-SREBP-1 complexes were sequentially incubated with SREBP-1 primary antibody (1:100) and HRP-conjugated anti-rabbit secondary antibody (1:100) for 1 h each at room temperature. Then, 100 μL of Transcription Factor Developing Solution was added into each well and the plate was incubated until adequate color development. Finally, the reaction was stopped by adding 100 μl of Stop Solution and the absorbance was read at 450 nm using a microplate reader.

### Transient transfection with small interfering RNA (siRNA)

To knock down genes of interest, cells were grown overnight to 50-70% confluency, followed by transfection with a target gene-specific siRNA or a scramble control siRNA using HiPerFect Transfection Reagent (#301704) from Qiagen (Hilden, Germany). The cells were then incubated for further 36 h before the transfection efficiency was examined by western blot analysis. The siRNA duplexes were synthesized by Bioneer (Daejeon, South Korea), and their sequences are shown in Table S1.

### RNA isolation and real time-quantitative polymerase chain reaction (RT-qPCR)

Total cellular RNA was extracted using Qiazol lysis reagent (#79306; Qiagen) according to the manufacturer’s instructions. Complementary DNA (cDNA) was subsequently synthesized from 1 μg of total RNA using GoScript™ Reverse Transcriptase (#A5000; Promega Corporation). qPCR was performed with ABsolute qPCR SYBR Green Mix using a Roche LightCycler 2.0 (Mannheim, Germany) as previously described [33]. The sense and antisense sequences of the primers used in this study are shown in Table S1.

### Western blot analysis

Cellular lysates were prepared from cells or tumor tissues using RIPA buffer (#89900; Thermo Scientific) containing 1X protease inhibitor cocktail (#78430; Thermo Scientific) and 1 mM PMSF (#36978; Thermo Scientific). Total protein in cellular lysate was measured using a BCA protein assay kit (#23227; Thermo Scientific). Equal amounts of protein were resolved on sodium dodecyl sulfate (SDS) polyacrylamide gel by electrophoresis, followed by transfer to PVDF membrane. The membrane was then blocked with 5% skim milk and sequentially incubated with a primary antibody (4 ^o^C, overnight) and an HRP-conjugated secondary antibody (room temperature, 1 h). The immunocomplexes were detected using an enhanced chemiluminescent (ECL) system (#34580; Thermo Scientific). Blot images were captured using the Fujifilm LAS-4000 mini (Fujifilm, Tokyo, Japan). Each blot shown in the figures is representative of at least three independent experiments, accompanied by a bar diagram that presents densitometric quantification of the blots.

### Immunoprecipitation (IP)

Cellular extracts were prepared from approximately 3 × 10^6^ cells using IP lysis buffer (#87787; Thermo Scientific). After removing nonspecifically bound cells by incubating the cellular lysates with protein G agarose (#20398; Thermo Scientific) for 1 h at 4 ^o^C, equal amounts of protein (500 μg) were incubated with a primary antibody (overnight at 4 ^o^C with gentle shaking). The immunocomplexes were pulled down by further incubation with protein G agarose for 4 h at 4 ^o^C. The beads were collected by centrifugation, washed twice with IP lysis buffer, treated with a denaturing buffer (containing 1X SDS and 2-mercaptoethanol), and heated (95 ^o^C for 10 min) to release the proteins of interest. Finally, the beads were removed and the supernatant was subjected to immunoblotting analysis.

### Immunofluorescence (IF) and confocal microscopic analysis

Cells were seeded into 8-well glass chamber slides at a density of 2-5 × 10^4^ cells/well. Lipid rafts were labeled in live cells using a Vybrant™ Alexa Fluor™ 488 Lipid Raft Labeling Kit (#V34403; Thermo Scientific). In principle, lipid raft domains were first labeled with Alexa Fluor 488-conjugated recombinant cholera toxin subunit B (CT-B) (1 μg/mL, 10 min on ice). Cells were then incubated with an anti-CT-B antibody (15 min on ice) to crosslink the CT-B-labeled lipid rafts into distinct patches on the cell membrane. If a cell surface protein labeling was required, cells were fixed with 4% paraformaldehyde for 15 min on ice, followed by sequentially incubating with a primary antibody (4 ^o^C, overnight) and an Alexa Fluor 594 conjugated anti-rabbit secondary antibody (#A-11012, Thermo Scientific).

For detection of colocalization between LC3 and lipid droplets, live cells were first incubated with Nile red as described above. Cells were next fixed with 4% paraformaldehyde, permeabilized with 0.2% Triton X-100, and sequentially incubated with an anti-LC3 primary antibody and an Alexa Fluor 488 conjugated anti-rabbit secondary antibody (#ab150077; Abcam). Next, cells were incubated with Bodipy 493/503, followed by further incubation with LysoTracker™ Red DND-99 (#L7528, Thermo Scientific) for 60 min under optimal culture conditions to investigate the overlap between lipid droplets and lysosomes. Before imaging, the slides were covered with a coverslip using ProLong™ Diamond Antifade Mountant with DAPI (#P36962; Thermo Scientific). Images were acquired using a confocal microscope A1 (Nikon, Tokyo, Japan).

### Development of orthotopic breast tumor model

All animal experiments were conducted according to the guidelines of the Yeungnam University Institutional Animal Care and Use Committee (IACUC). The animal protocols were reviewed and approved by the Yeungnam University IACUC (Protocol number: 2021-009). For establishment of orthotopic breast tumors, 8-week-old female BALB/c nude mice (Orient Ltd., Osan, South Korea) were anesthetized by intraperitoneal injections of xylazine/ketamine (10 and 100 mg/kg, respectively). After the fourth mammary fat pad was exposed by making a small incision between the middle and the fourth nipple, MDA-MB-231-luc cells (10^6^ cells) in a 1:1 mixture of fresh media and Matrigel (#356321; Corning Inc., Corning, NY, USA) were injected into the mammary fat pad. Once the xenograft had reached about 50 mm^3^, mice were randomly allocated into four groups (n=5) and given one of the following treatments: gAcrp (1 μg/tumor/day, peritumoral injection), [7]; EX527 (2 mg/kg/day, i.p) [34], gAcrp plus EX527, and PBS (control). During treatment period, the tumor growth was monitored twice a week by measuring bioluminescence signal intensity upon intraperitoneal injection of luciferase substrate D-luciferin (150 mg/kg, i.p) using IVIS® Lumina III In Vivo Imaging System (PerkinElmer Inc., Waltham, MA, USA). After 4 weeks of treatment, the mice were sacrificed and tumors were excised, weighed, and stored for further analyses.

### Immunohistochemistry (IHC)

IHC was carried out according to the previously described method [32]. Briefly, tumor sections (10-μm thick) were prepared from paraformaldehyde-fixed tissues using a freezing sliding microtome (Microm HM 450, Thermo Scientific). After mounting onto gelatin-coated glass slides, the sections were treated with EDTA buffer (pH 8) at 95 ^o^C for 10 min for antigen retrieval, followed by sequential incubation with 3% H_2_O_2_ and 5% normal goat serum for blocking endogenous peroxidase activity and nonspecific binding. The sections were then incubated with a primary antibody (4 ^o^C, overnight) and a biotinylated secondary antibody (room temperature, 90 min). The resultant immunocomplexes were detected using peroxidase-based detection system (#PK-6100, Vector Laboratories Inc.). Images were captured using a light microscope (BX41 TF, Olympus, Tokyo, Japan) and analyzed by the open-source platform Image J [35].

### Statistical analysis

Data are expressed as the mean ± standard error of the mean (SEM) from at least three independent experiments and analyzed using GraphPad Prism 8.02 (San Diego, CA, USA). Significant differences were examined using one-way analysis of variance (ANOVA) or t-test (if only two conditions were used). Statistical significance was set at p-value < 0.05.

## Results

### Adiponectin decreases cellular lipid pool in breast cancer cells

**Fig. 1:**
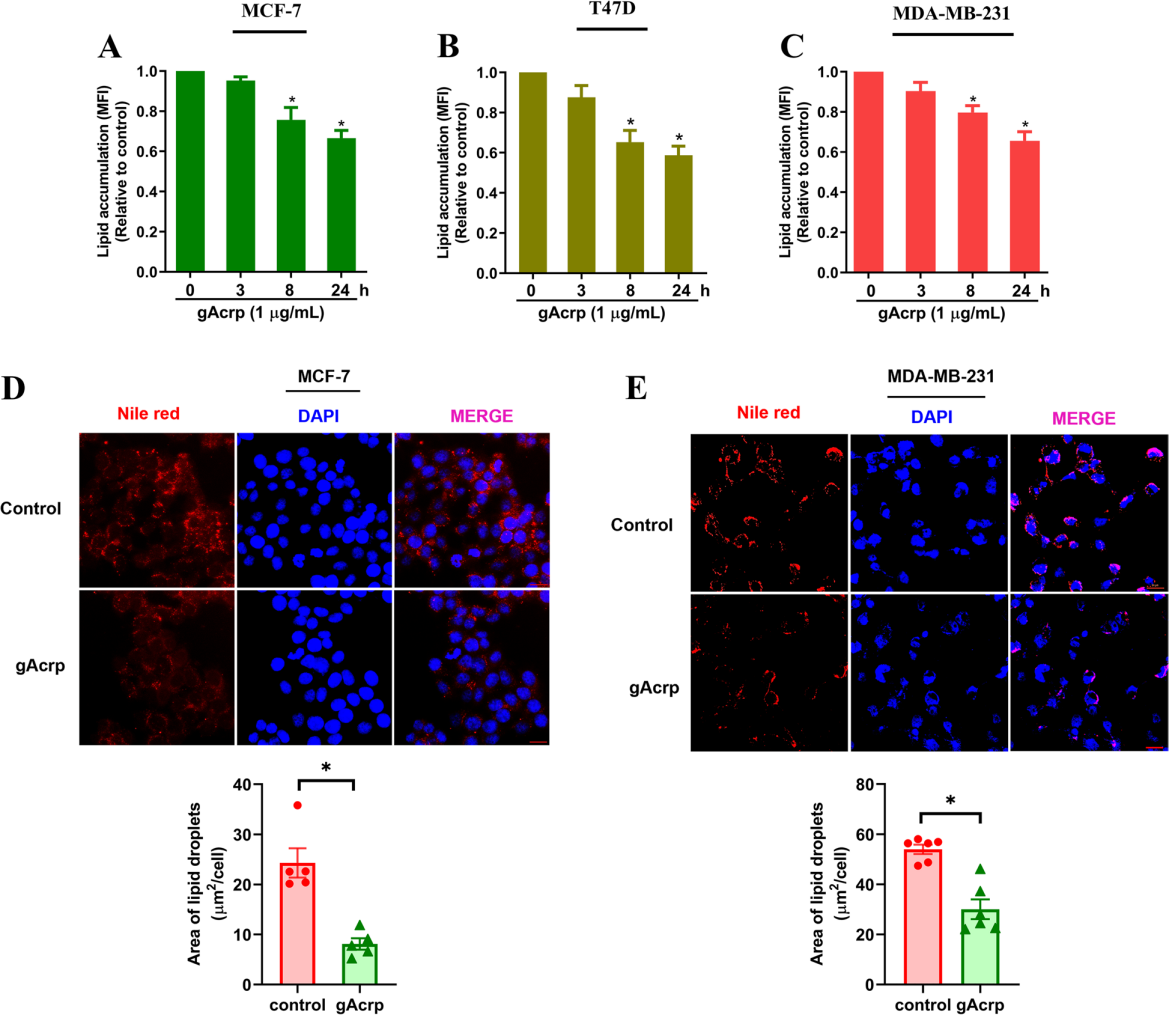
Effect of globular adiponectin on cellular lipid reservation in breast cancer cells. **A-C** MCF-7 (**A**), T47D (**B**), and MDA-MB-231 (**C**) cells were treated with gAcrp (1 μg/mL) for indicated time periods. Cells were then incubated with Bodipy 493/503 for 30 min. Neutral lipid accumulation was determined as mean fluorescence intensity (MFI) using flow cytometry analysis. **D-E** MCF-7 (**D**) and MDA-MB-231 (**E**) cells were treated with gAcrp for 24 h, followed by labeling of lipid droplets with Nile red. Representative images for gAcrp-treated and control cells were shown along with quantification of lipid droplet area in respect to DAPI, in which the lipid droplet areas were determined by total area of red fluorescence using Image J software (scale bar: 20 μm). * denotes p < 0.05 compared to control cells (n=3 except where specifically indicated in Figures)

To investigate if adiponectin affects lipid metabolism in breast cancer cells, we first examined its effects on cellular lipid pool. Using Bodipy 493/503 as a fluorescent probe of neural lipids, we found that globular adiponectin (gAcrp) decreased Bodipy 493/503 uptake in MCF-7 cells in a time-dependent manner (Fig. 1A), indicating that adiponectin impairs cellular lipid reservation. This effect was also observed in different types of breast cancer cells, including T47D (Fig. 1B) and triple-negative MDA-MB-231 cells (Fig. 1C). Impaired intracellular lipid accumulation was further confirmed by confocal microscopic images showing that gAcrp treatment led to drastic reduction in the number of lipid droplets in both MCF-7 (Fig. 1D) and MDA-MB-231 (Fig. 1) cells, as determined by Nile red staining. Together, these findings clearly show that adiponectin induces a reduction in lipid content in both ER-positive and -negative breast cancer cells.

### Adiponectin-induced lipid depletion triggers lipid raft disruption and apoptosis in breast cancer cells

**Fig. 2:**
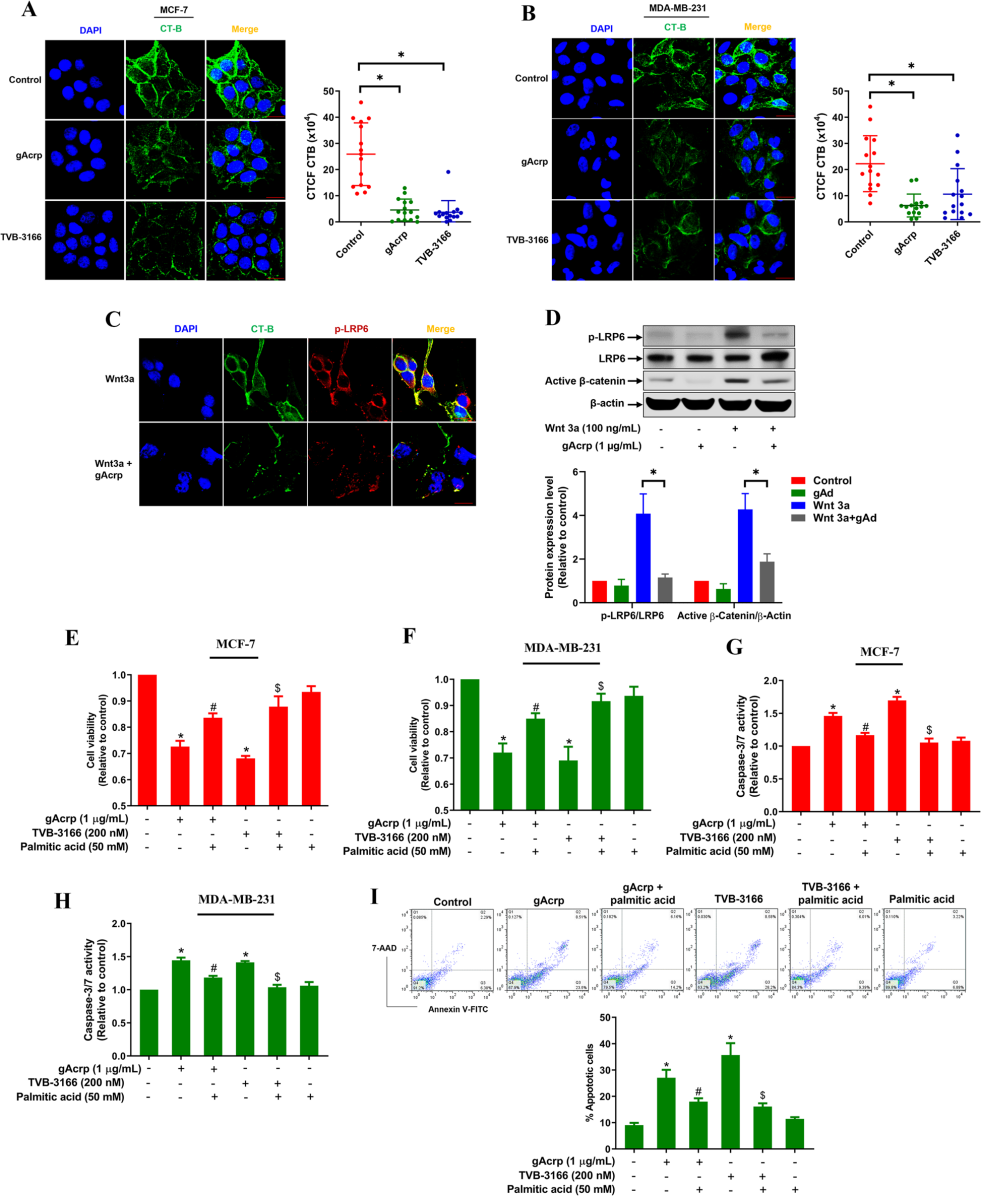
Critical roles of lipid metabolic remodeling in breast cancer cell death induction by globular adiponectin. **A-B** MCF-7 (**A**) and MDA-MB-231 cells (**B**) were treated with gAcrp (1 μg/mL) or TVB-3166 (200 nM), a pharmacological inhibitor of FASN, for 48 h. Lipid raft microdomains were labeled with Alexa fluor 488-conjugated Cholera toxin B (CT-B) as described in Methods (green color). Representative images from three independent experiments were presented (upper panel) along with quantification of the corrected total cell fluorescence (CTCF) for CT-B (lower panel), in which CTCF was determined by integrated density - (area of selected cell × mean fluorescence of background readings) as previously described [38]. **C-D** MDA-MB-231 cells were treated with gAcrp for 48 h, followed by further stimulated with Wnt3a (100 ng/mL) for 6 h. **C** Lipid raft (green) was stained with Alexa fluor 488-conjugated CT-B and membrane p-LRP6 was labeled with a specific primary antibody and an Alexa fluor 594-conjugated secondary antibody (red). Scale bar: 20 μm. **D** p-LRP6, LRP6, and non-phospho- (active) β-catenin levels were examined by western blot analysis. Bar diagram shows quantification of blots. **E-I** MCF-7 (**E** and **G**) and MDA-MB-231 (**F**, **H**, and **I**) cells were treated with gAcrp (1 μg/mL) or TVB-3166 (200 nM) for 48 h in serum-free culture media in the absence of presence of BSA-conjugated palmitic acid (50 mM). Cell viability (**E**-**F**) and capase-3/7 activity (**G**-**H**) were determined at the end of treatment period as described in Methods. **I** Cells were double-stained with FITC-annexin V and 7-AAD, and subjected to flow cytometry analysis. Bar diagram shows the percentage of annexin V-positive cells that represents apoptotic cell population. * indicates p < 0.05 compared to control cells; # indicates p < 0.05 compared to cells treated with gAcrp alone; $ indicates p < 0.05 compared to cells treated with TVB-3166 alone; n=3 except where specifically indicated in Figures

Lipid molecules play a critical role in the regulation of membrane architecture and membrane protein-mediated signal transduction. It has been previously reported that lipid rafts, which are cholesterol and sphingolipid-rich membrane microdomains serving as sorting platforms for signal transduction molecules, are highly sensitive to alterations in cellular lipid homeostasis [36]. Since adiponectin induces a lipid depletion in breast cancer cells, we investigated whether it disrupts membrane lipid raft structure. By fluorescent labeling of lipid rafts with cholera toxin-B (CT-B), we observed that CT-B staining significantly decreased upon long-term treatment with gAcrp in MCF-7 (Fig. 2A) and MDA-MB-231 (Fig. 2B) cells. This effect was essentially similar to that of TVB-3166, a FASN inhibitor, suggesting that lipid depletion by adiponectin causes loss of lipid raft microdomains in breast cancer cells. Given the role of lipid rafts as signaling scaffolds for various receptors, we next sought to verify the effect of lipid raft disorganization on raft-dependent activation of certain signaling molecules. As a typical example, phosphorylation of LRP6, a coreceptor for Wnt and essentially required for canonical Wnt/β-catenin signaling pathway, has been reported to occur only in lipid raft domains [37]. Herein, while there was a high degree of colocalization between p-LRP6 and lipid rafts in the presence of Wnt3a ligand, the loss of integrity of raft architecture by adiponectin was accompanied by a dramatic reduction in the expression of p-LRP6 on the plasma membrane (Fig. 2C). Impaired response to the Wnt ligand was further confirmed by western blot analysis, in which gAcrp restored the levels of p-LRP6 and active β-catenin in Wnt3a-stimulated MDA-MB-231 cells (Fig. 2D). Furthermore, we found that gAcrp treatment led to long-term inactivation of Akt (Supplementary figures, Fig. S1A and S1B), another signal molecule whose phosphorylation has been documented to be lipid raft dependent. These findings reveal that lipid depletion by adiponectin induces lipid raft disruption and inhibition of transmembrane signal transduction.

Having shown that decreased cellular lipid content by adiponectin causes disorders of in the structures and function of lipid raft, we speculated that the alterations in lipid metabolism may contribute to the anti-breast cancer effects of adiponectin. We first confirmed that gAcrp and TVB-3166 reduced the viability of MCF-7 and MDA-MB-231 cells (Fig. S2A-S2D). Interestingly, decreased cell survival by adiponectin and TVB-3166 was significantly restored by supplementation of the culture media with bovine serum albumin (BSA)-conjugated palmitic acid (Fig. 2E and F). In addition, exogenous palmitate also rescued breast cancer cells from adiponectin- and TVB-3166-induced apoptotic death, as determined by caspase-3/7 activity assay (Fig. 2G and H) and annexin V staining (Fig. 2I). Taken together, these results imply that dysregulated cellular lipid homeostasis is responsible, at least in part, for the induction of breast cancer cell death by adiponectin.

### Adiponectin suppresses fatty acid synthesis via downregulation of SREBP-1 in breast cancer cells

**Fig. 3:**
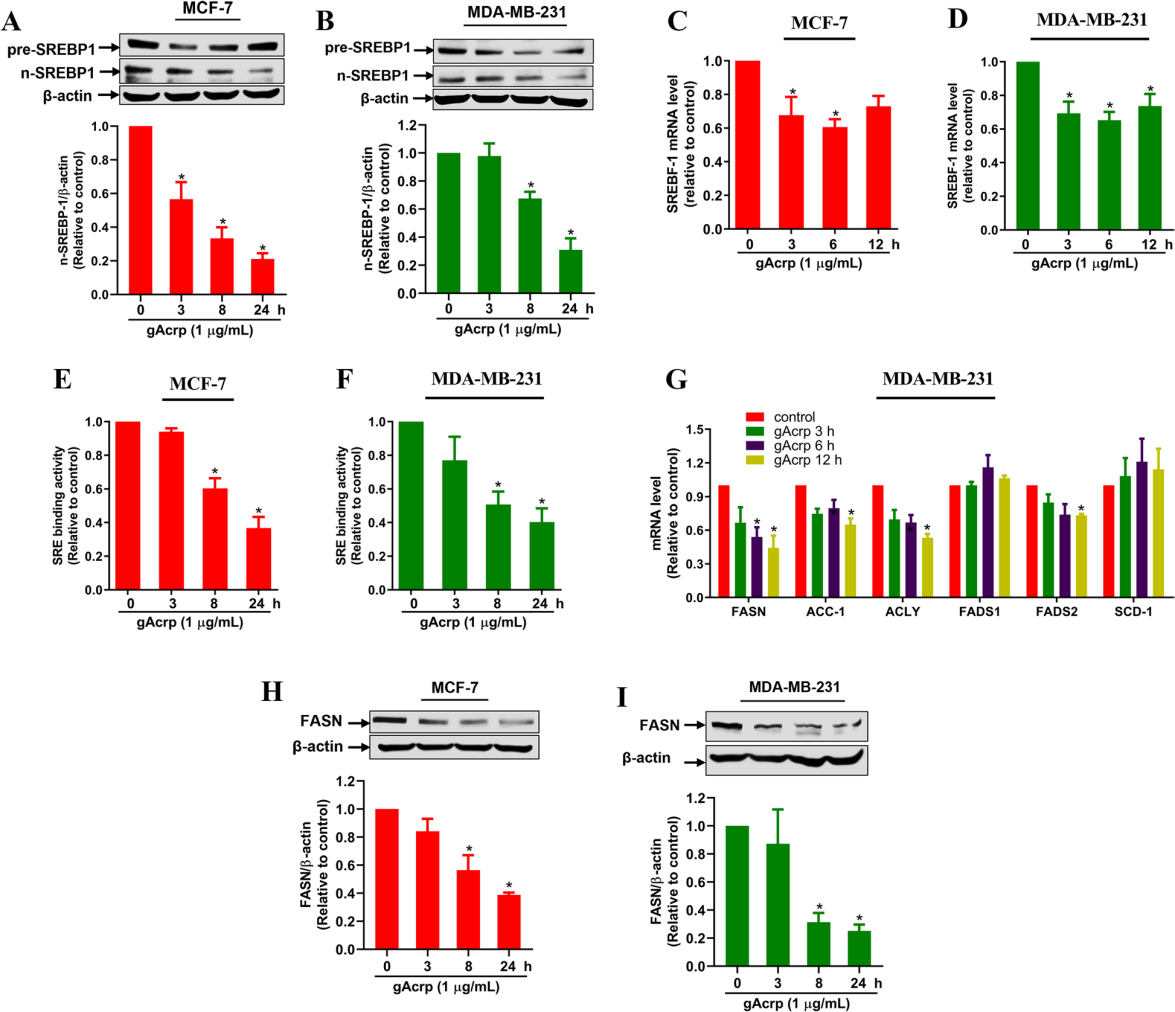
Modulation of fatty-acid-synthesis related genes by globular adiponectin in breast cancer cells. MCF-7 (A, C, E, and H) and MDA-MB-231 (B, D, F, G, and I) cells were treated with gAcrp (1 μg/mL) for indicated time periods. **A-B** The expression levels of precursor and nuclear SREBP-1 were determined by western blot analysis. **C-D** SREBP-1 mRNA levels were measured by RT-qPCR. **E-F** The SREBP-1 specific dsDNA binding activity was examined as described in Methods. **G** The expression of the genes modulating fatty acid synthesis, including FASN, ACC-1, ACLY, FADS1, FADS2, and SCD-1, were analyzed by RT-qPCR. **H-I** The protein levels of FASN were examined by western blot analysis. * indicates p < 0.05 compared to control; n=3 except where specifically indicated in Figures

Impaired cellular lipid pool may result from reduced biosynthesis, increased degradation, or decreased lipid uptake. Considering the importance of *de novo* FAS in cancer cell survival and proliferation, we next examined the effect of adiponectin on the expression of SREBP-1 and FAS-modulating genes. As shown in Fig. 3, gAcrp significantly suppressed the expression of nuclear SREBP-1 in a time-dependent manner, accompanied by a transient decrease in the respective precursor form (Fig. 3A and B). Consistently, gAcrp downregulated SREBP-1 mRNA expression (Fig. 3C and D) and also decreased the binding of SREBP-1 in nuclear extract to a specific dsDNA sequence containing the SREBP-1 response element (SRE) (Fig. 3E and F), clearly indicating that adiponectin suppressed the transcriptional activity of SREBP-1 in breast cancer cells. As SREBP-1 is a master regulator of FAS-modulating genes, we investigated downstream effects of adiponectin on SREBP-1 suppression. Among the target genes of SREBP-1, gAcrp prominently suppressed the mRNA expression of FASN and decreased the mRNA levels of ACC-1, ACLY, and FADS2 in relatively smaller degrees without significant effects on FADS1 and SCD-1. Downregulation of FASN by adiponectin was further confirmed by western blot analysis (Fig. 3H and I). Together, these results suggest that adiponectin decreases FAS levels by suppressing SREBP-1 and its downstream targets.

### Adiponectin induces lipolysis through activation of lipophagy in breast cancer cells

**Fig. 4:**
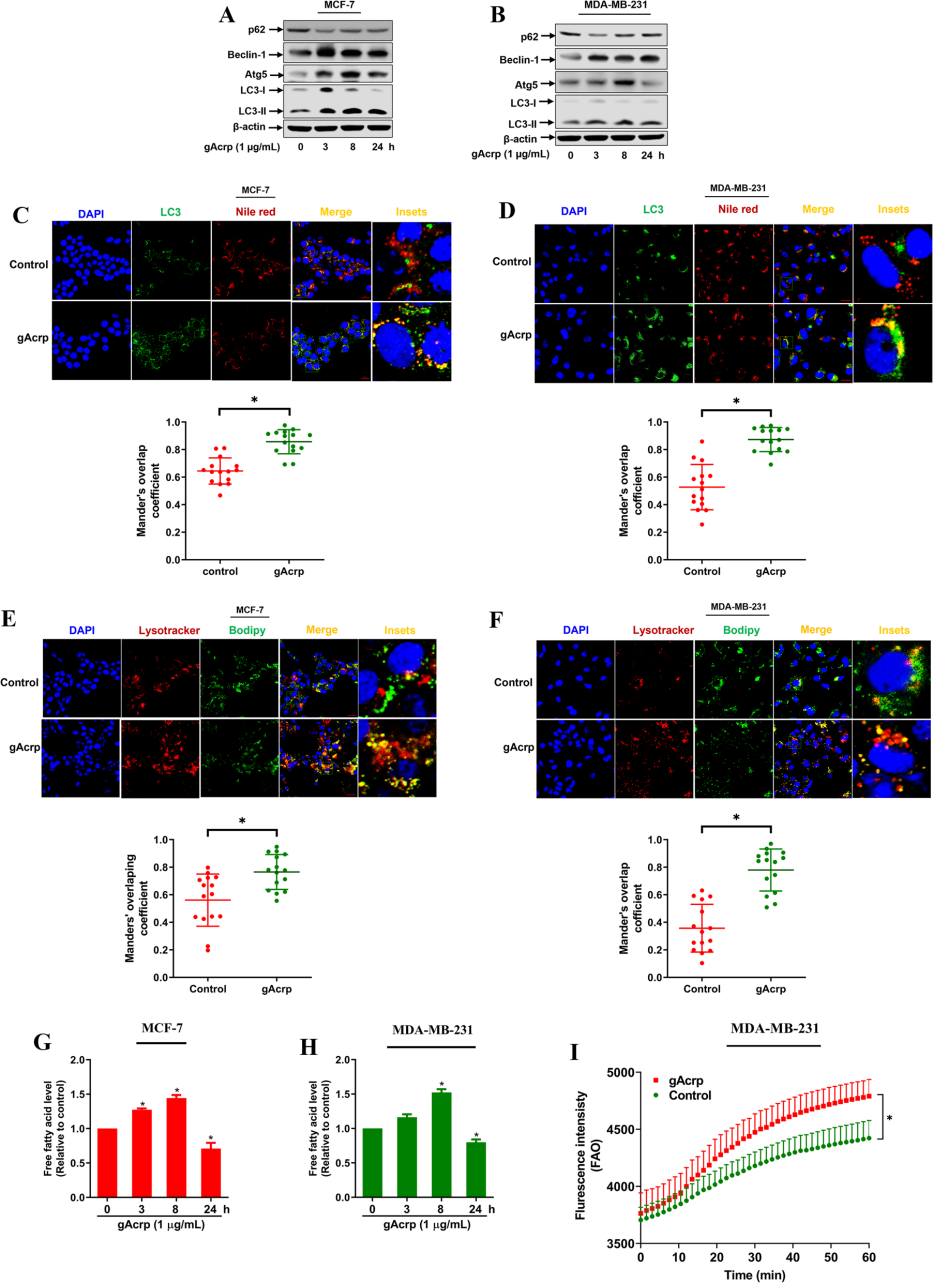
Activation of lipophagy by globular adiponectin in breast cancer cells. **A-B** MCF-7 (**A**) and MDA-MB-231 (**B**) cells were treated with 1 μg/mL of gAcrp for different time periods. The expression levels of autophagy-related genes, including p62, Beclin-1, Atg5, and LC3I/II were examined by western blot analysis. **C-F** Cells were cultured in the media containing 10 μM of oleic acid/BSA to induce lipid droplet formation and treated with 1 μg/mL of gAcrp for 6 h. **C**-**D** Lipid droplets and LC3 were detected by labelling with Nile red (red), and incubation with an anti-LC3 primary antibody followed by Alexa fluor 488-conjugated antibody (green), respectively. **E**-**F** Lipid droplets and lysosomes were labeled with Bodipy 493/503 (green) and Lysotracker (red). Co-localization rate was determined by Mander’s overlap coefficient using Image J software. Scale bar: 20 μm. **G-H** Cells were treated with gAcrp for the indicated time duration. Free fatty acid levels were measured at the end of treatment periods as indicated in the methods. **I** MDA-MB-231 cells were treated with gAcrp (1 μg/mL) for 8 h. Fatty acid oxidation (FAO) was determined as described in the methods. * denotes p < 0.05 compared to control cells; n=3 except where specifically indicated in Figures

Autophagy has been postulated to play a key role in the regulation of lipid metabolism [39]. Moreover, autophagy activation has been recently proposed as a molecular mechanism underlying the breast cancer-suppressing effect of adiponectin [4]. Therefore, we assumed that autophagy is implicated in alterations in lipid metabolism by adiponectin. We first confirmed that gAcrp induced activation of autophagy in MCF-7 and MDA-MB-231 cells, as evidenced by increased LCI/LC3-II conversion, enhanced expression of Beclin-1 and Atg5, and reduced p62 levels (Fig. 4A and B). To examine if adiponectin promotes autophagic degradation of lipid droplets, we labeled autophagosomes and lipid droplets with anti-LC3 antibody and Nile red, respectively. As shown in Fig. 4C and D, there was a remarkable increase in the colocalization of LC3 and Nile red after gAcrp treatment, suggesting that adiponectin promotes the delivery of lipid droplets to autophagic vacuoles. The enhanced interaction between autophagic vacuoles and lipid droplets by adiponectin was further confirmed by overlap between Lysotracker Red (lysosomes) and Bodipy 493/503 (lipid droplets) (Fig. 4E and F). Next, we examined the effect of gAcrp on cellular FFA, one of the main products of lipid droplet degradation. Intriguingly, while a significant increase in free fatty acid level was observed at early phase (up to 8 h), this level markedly decreased upon long-term treatment (at 24 h) of gAcrp (Fig. 4G and H). Given the role of FFA as an energy substrate, we finally verified if activation of lipolysis by adiponectin leads to increased FAO. As expected, gAcrp promoted FAO in breast cancer cells (Fig. 4I), suggesting that stimulation of lipolysis by adiponectin was coupled with FAO. Collectively, these findings demonstrate that adiponectin, through activation of lipophagy, induces the release of FFA which is subsequently degraded by FAO.

### Lipid metabolic remodeling by adiponectin in breast cancer cells is mediated via SIRT-1 induction

**Fig. 5:**
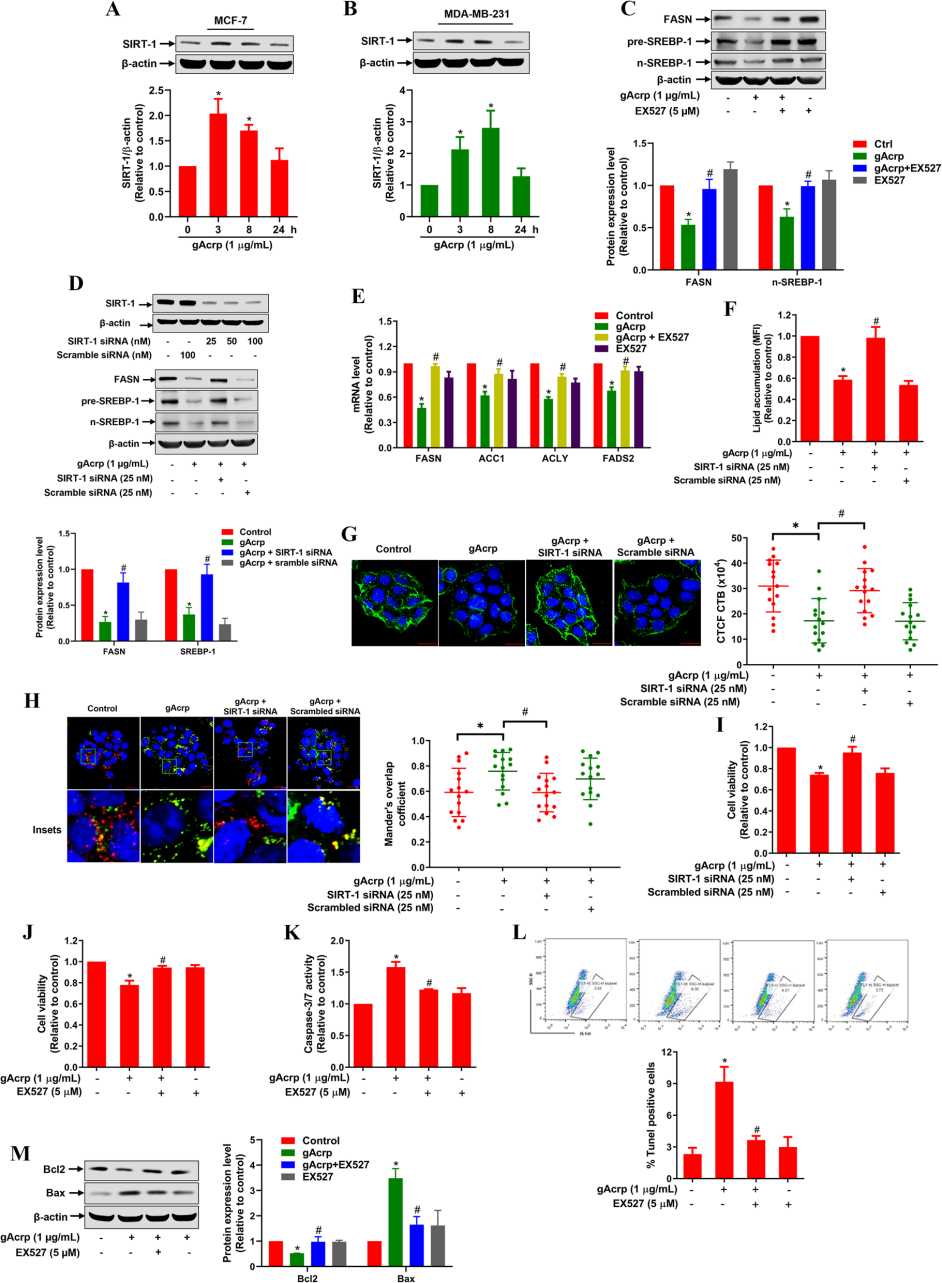
Roles of SIRT-1 in fatty acid metabolic reprogramming by globular adiponectin in breast cancer cells. **A-B** MCF-7 (**A**) and MDA-MB-231 (**B**) cells were treated with gAcrp as indicated. SIRT-1 expression was determined by western blot analysis. **C** MCF-7 cells were pretreated with EX527 (5 μM) for 2 h, followed by incubation with gAcrp (1 μg/mL) for further 8 h. The protein levels of SREBP-1 and FASN were examined by western blot analysis. **D** MCF-7 cells were transfected with a siRNA targeting SIRT-1 or a scramble control siRNA for 36 h, followed by treatment with gAcrp (1 μg/mL) for 8 h. (Upper panel) The gene silencing efficiency was monitored by western blot analysis. (Lower panel) The expression levels of FASN and SREBP-1 in SIRT-1 knockdown cells were examined after gAcrp treatment. **E** MCF-7 cells were pretreated with EX527 for 2 h, followed by further incubation with gAcrp (1 μg/mL) for 12 h. mRNA levels of FASN, ACC-1, ACLY, and FADS2 were measured by RT-qPCR. **F-H** MCF-7 cells were transfected with SIRT-1 siRNA (25 nM) for 36 h, followed by treatment with 1 μg/mL of gAcrp for 24 h (**F**), 48 h (**G**), or 6 h (**H**). **F** Cellular neutral lipid content was determined by Bodipy 493/503 uptake assay. **G** Cells were labeled with Alexa fluor 488-conjugated CT-B and lipid raft microdomains were observed under a confocal microscope. **H** Lipid droplets were stained with Nile red and autophagosomes were labeled with an Alexa fluor 488-conjugated anti-LC3 antibody. The overlapping between lipid droplets (red) and autophagosomes (green) were observed under a confocal microscope. The Mander’s overlap coefficient was used to test the colocalization of lipid droplets and autophagosomes. Scale bar: 20 μm. **I-M** MCF-7 cells were transfected with SIRT-1 siRNA (25 nM) (**I**) or pretreated with EX527 for 2 h (**J**-**M**), followed by further treatment with 1 μg/mL of gAcrp for 48 h. **I** and **J** Cell viability was measured by MTS assay. **K**-**L** The apoptosis level was determined using caspase-3/7 activity (**K**) and TUNEL assay (**L**) as indicated in the methods. **M** Expression levels of Bax and Bcl2 were examined by western blot analysis. * denotes p < 0.05 compared to control; # denotes p < 0.05 compared to cells treated with gAcrp alone; n=3 except where specifically indicated in Figures

SIRT-1, acts as a master cellular metabolic regulator, has been reported as a downstream effector of adiponectin [31]. To elucidate the molecular mechanisms underlying lipid metabolic reprogramming by adiponectin, we examined the involvement of SIRT-1 signaling in adiponectin-modulated lipid metabolism. As expected, gAcrp induced SIRT-1 expression in MCF-7 and MDA-MB-231 cells in a time-dependent manner (Fig. 5A and B). SIRT-1 induction by gAcrp was demonstrated to be dependent on ROS production (Fig. S3A and S3B). Importantly, inhibition of SIRT-1 by treatment with its pharmacological inhibitor (EX527) or transfection with siRNA targeting SIRT-1 abolished the suppressive effect of gAcrp on SREBP-1 and FASN expression (Fig. 5C and D). Likewise, mRNA levels of FAS genes, including FASN, ACC-1, FADS2, and ACLY, were restored by pretreatment with EX527 (Fig. 5EE), indicating a critical role of SIRT-1 induction in adiponectin-modulation of FAS. It is also notable that blockage of SIRT-1 prevented lipid depletion (Fig. 5F), lipid raft disruption (Fig. 5G), and lipophagy activation (Fig. 5H) by gAcrp, further confirming that SIRT-1 drives lipid metabolic reprogramming in adiponectin-stimulated breast cancer cells. Given the previous findings that altered lipid metabolism critically contributes to the induction of breast cancer cell death by adiponectin, subsequent experiments were designed to determine the implication of SIRT-1 in the modulation of cancer cell fate. The results showed that decreased cell viability by gAcrp was mostly restored by pretreatment with EX527 or SIRT-1 knockdown (Fig. 5I and J). In addition, pretreatment with EX527 rescued breast cancer cells from apoptosis by adiponectin, as determined by caspase-3/7 activity (Fig. 5K) and TUNEL (Fig. 5L) assays. Moreover, while gAcrp was found to promote apoptosis through enhanced Bax expression and decreased Bcl2 level, these modulatory effects were antagonized by EX527 (Fig. 5M). Together, these results reveal that SIRT-1 induction is essential for lipid metabolic reprogramming and apoptosis induction by adiponectin.

### SIRT-1 contributes to fatty acid metabolic alterations by adiponectin via deacetylation of SREBP-1 and suppression of mTOR

**Fig. 6:**
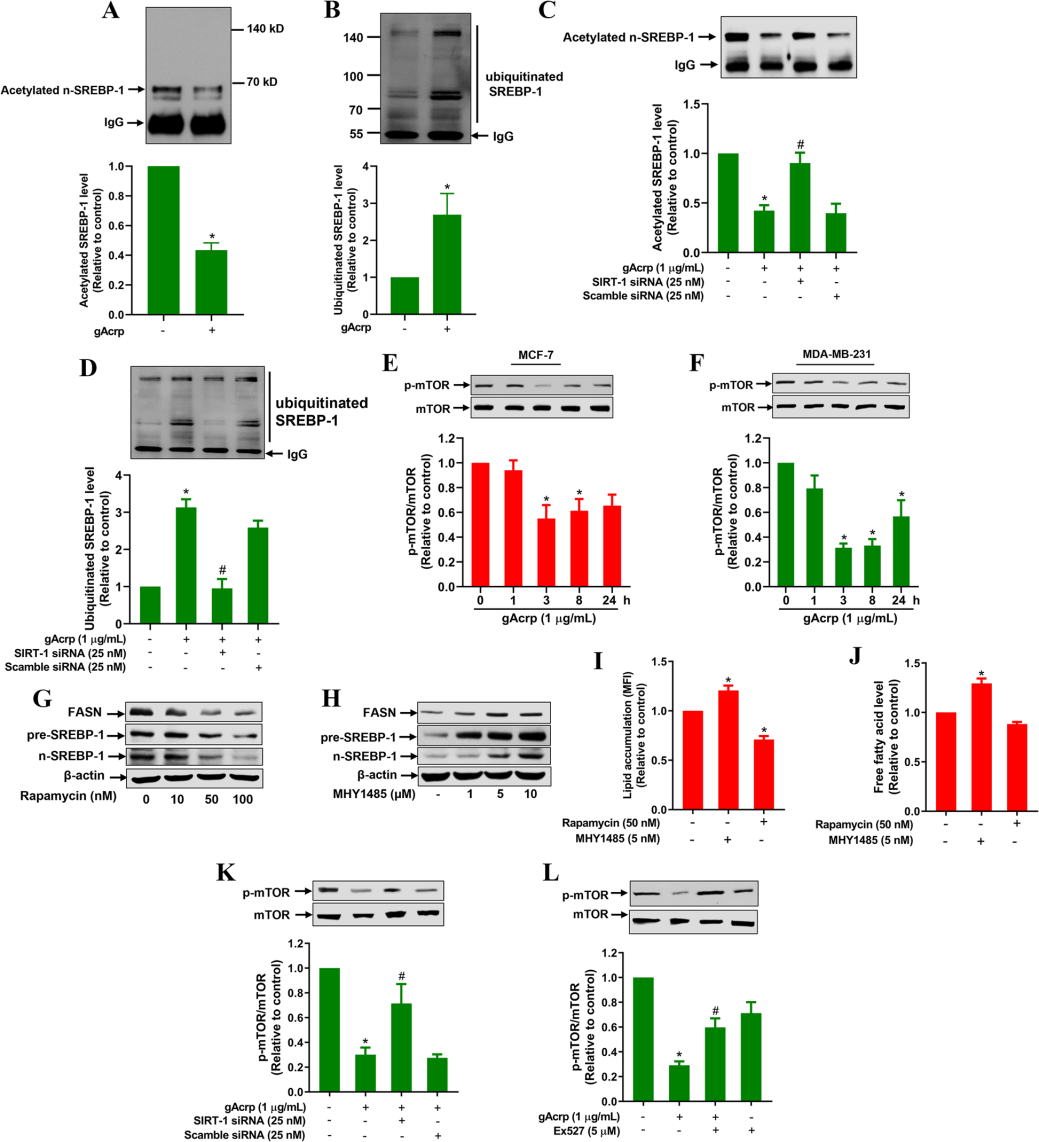
Involvement of SIRT-1 induction and mTOR signaling in the modulation of SREBP-1 by globular adiponectin. **A-D** MCF-7 cells were treated with gAcrp (1 μg/mL) for 8 h (**A**-**B**) or transfected with 25 nM SIRT-1 siRNA for 36 h before treatment with gAcrp for 8 h. (**A** and **C**) Acetylated SREBP-1 level was determined by immunoprecipitation with an anti-acetyl lysine antibody and western blot analysis using an anti-SREBP-1 primary antibody. **B** and **D** Ubiquitinated SREBP-1 level was measured by immunoprecipitation with an anti-ubiquitin antibody, followed by western blot analysis using an anti-SREBP-1 primary antibody. **E-F** Cells were treated with gAcrp (1 μg/mL) for different time periods. The total and phospho-mTOR levels were determined by immunoblotting. **G-H** MCF-7 cells were transfected with SIRT-1 siRNA for 36 h (**G**) or pretreated with EX527 for 2 h (**H**), followed by treatment with gAcrp for additional 3 h. p-mTOR/mTOR levels were examined by western blot analysis. **I-J** MCF-7 cells were treated with different concentrations of rapamycin (**I**) and MHY1485 (**J**) for 8 h. SREBP-1 and FASN expression levels were measured by western blot analysis. **K-L** MCF-7 cells were treated with rapamycin and MHY1485 for 24 h. Intracellular lipid accumulation (**K**) and free fatty acid (**L**) levels were determined as described above. * indicates p < 0.05 compared to control; # indicates p < 0.05 compared to gAcrp-treated cells; n=3

Having demonstrated that SIRT-1 mediates the metabolic actions of adiponectin, we next sought to elucidate the mechanisms by which SIRT-1 induction drives cellular fatty acid metabolism. As a deacetylase, SIRT-1 has been reported to regulate SREBP-1 acetylation [40]. Hence, we speculated that adiponectin modulates acetylation of SREBP-1 via SIRT-1 induction. Therefore, we measured the acetylated levels of SREBP-1 in MCF-7 cells by immunoprecipitation assay and found that gAcrp markedly reduced the level of acetylated nuclear SREBP-1 (Fig. 6A). Interestingly, decreased SREBP-1 acetylation was accompanied by enhanced ubiquitinylated SREBP-1 level (Fig. 6B), suggesting that deacetylation of SREBP-1 by adiponectin caused instability and promoted degradation of SREBP-1 through the ubiquitin pathway. The role of SIRT-1 in deacetylation and degradation of SREBP-1 was confirmed in subsequent experiments, which showed that SIRT-1 knockdown abrogated the effect of gAcrp on acetylation and ubiquitination of SREBP-1 (Fig. 6C and D).

SREBP-1 expression is modulated via multiple mechanisms, including post-translational regulation involving acetylation. To further elucidate the underlying molecular mechanisms, we examined the involvement of mTOR, which has been known as a modulator of SREBP-1 expression in various cell types [41]. We found that gAcrp significantly suppressed phosphorylation of mTOR in MCF-7 (Fig. 6E) and MDA-MB-231 (Fig. 6F) breast cancer cells. The stimulatory role of mTOR signaling in SREBP-1 expression in breast cancer cells was confirmed by demonstrating that rapamycin, a pharmacological inhibitor of mTOR, markedly decreased SREBP-1 expression (Fig. 6G), whereas activation of mTOR by treatment with MHY1485 enhanced expression levels of SREBP-1 (Fig. 6H). Essentially similar effects on FASN expression were observed (Fig. 6G and H). Consistently, MHY1485 caused significant accumulation of neutral lipids and enhanced cellular free fatty acid levels, whereas rapamycin generates opposite effects on cellular lipid content in MCF-7 cells (Fig. 6I and J), collectively indicating the crucial roles of mTOR signaling in SREBP-1 expression and lipid metabolism in breast cancer cells treated with adiponectin. Interestingly, inhibition of SIRT-1 by transfection with a specific siRNA or EX527 partly abrogated suppression of mTOR phosphorylation by gAcrp (Fig. 6K and L), suggesting that SIRT-1 signaling is also implicated in the suppression of mTOR activation by adiponectin. Taken together, these results here demonstrate that induction of SIRT-1 by adiponectin modulates SREBP-1 expression and activity either by direct deacetylation of its mature form or indirectly via suppression of mTOR.

### Adiponectin modulates fatty acid metabolic reprogramming and tumor growth in vivo orthotopic breast tumors

**Fig. 7:**
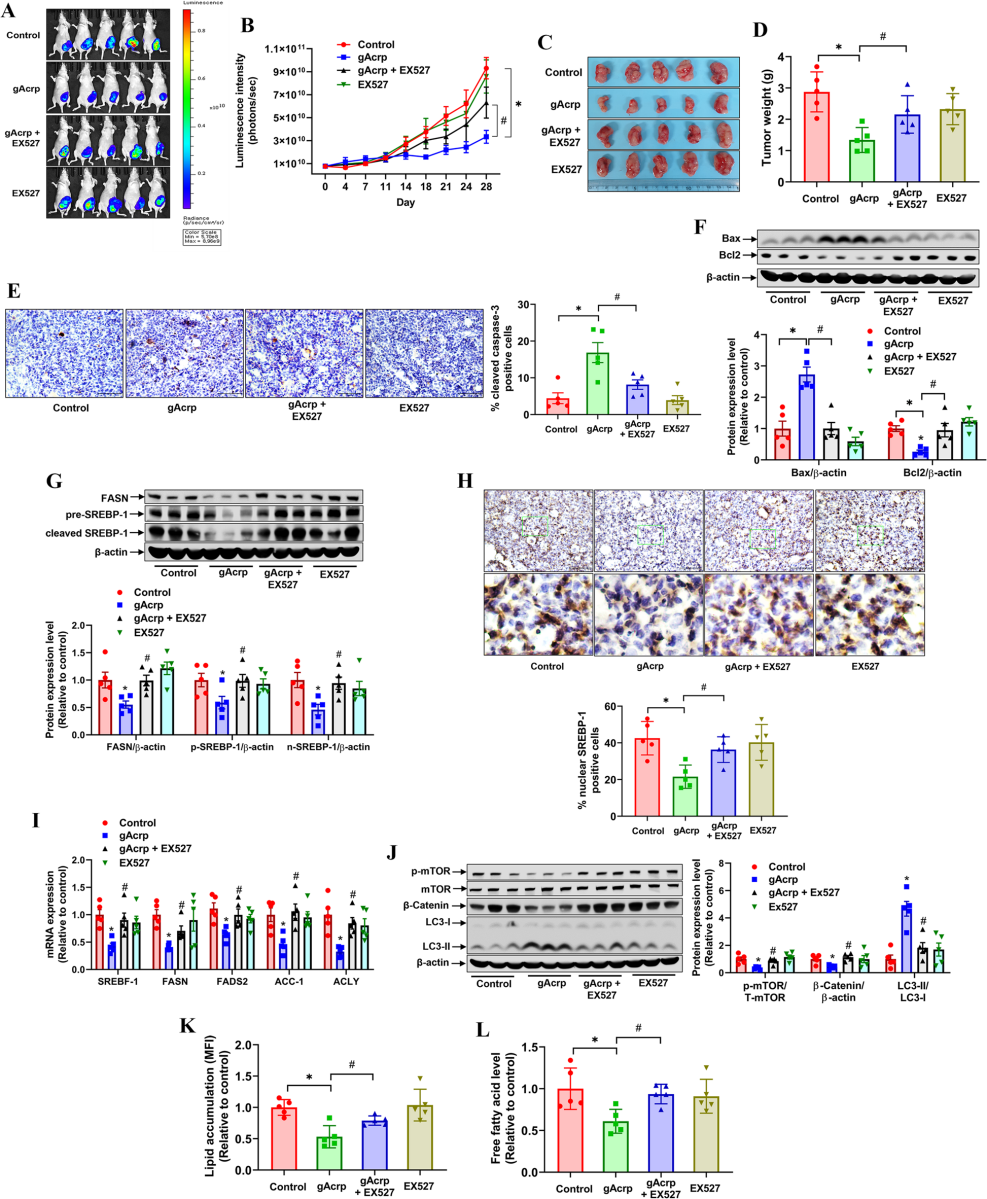
Role of SIRT-1 in adiponectin modulation of *in vivo* breast tumor lipid metabolism and growth. MDA-MB-231-luc orthotopic breast tumor**s** were generated in BALB/c nude mice, followed by treatment with gAcrp alone or gAcrp in combination with EX527 for 28 days. **A **and** B** Luminescent images of tumors (**A**) and tumor growth rate were monitored by luminescent *in vivo* imaging during treatment (**B**). **C **and** D** Tumor tissues were harvested after 4 weeks of treatment. Isolated tumors were captured at the end of experiment (**C**) and tumor weight was recorded (**D**). **E** Tissue section was prepared, and cleavage of caspase-3 was examined by immunohistochemistry (IHC). The percentage of cleaved caspase-3 positive tumor cells was determined by Image J software. **F** The expression levels of Bax and Bcl2 were measured by western blot analysis. The representative images from 3 mice each group were shown along with blot quantification for all collected tumor tissues. **G** The expression levels of FASN and SREBP-1 were analyzed by immunoblotting analysis. **H** SREBP-1 was detected in tumor tissues by IHC. The proportion of nuclear SREBP-1 positive cells were presented in bar diagram. Scale bar: 100 μm. **I** The mRNA levels of SREBP-1, FASN, ACC-1, FADS2, and ACLY in tumor tissues were measured by RT-qPCR. **J** The protein levels of p-mTOR, mTOR, β-catenin, and LC3I/II were determined by western blot analysis. **K-L** Single cells were isolated from tumor tissues by incubating with collagenase solution. **K** Tumor cells were incubated with Bodipy 493/503 for 15 min at 37^o^C, followed by flow cytometry analysis. **L** The free fatty acid level was measured in tumor cells and normalized to tumor cell number

To validate the modulatory effects of adiponectin on cellular fatty acid metabolism and the role of SIRT-1 signaling in its metabolic and anti-cancer actions under *in vivo* conditions, an orthotopic breast tumor model was established in BALB/c nude mice and the mice bearing breast tumors were treated with gAcrp and/or a pharmacological inhibitor of SIRT-1 (EX527). We first confirmed the SIRT-1 inducing effect of adiponectin in tumor tissues by western blot analysis (Fig. S4A) and IHC (Fig. S4B). As expected, EX527 abrogated the suppressive effect of gAcrp on tumor growth as measured by bioluminescence imaging and tumor weight (Fig. 7A-D). In addition, co-treatment with EX527 markedly reduced the proportion of cleaved caspase-3 positive cells (Fig. 7E) and Bax expression, but enhanced Bcl2 levels (Fig. 7F) in gAcrp-treated tumors, indicating that inhibition of SIRT-1 prevented tumor cells from apoptosis by adiponectin. In line with *in vitro* observations, gAcrp downregulated SREBP-1 (Fig. 7G) and suppressed the expression of nuclear SREBP-1 (Fig. 7H) in tumor tissues. Moreover, gAcrp was found to decrease mRNA levels of SREBP-1 and FAS genes, including FASN, ACC1, FADS2, and ACLY (Fig. 7I). Downregulation of FASN was further validated by immunoblotting (Fig. 7G). In addition, gAcrp decreased the expression levels of p-mTOR and active β-catenin but increased LC3-II levels (Fig. 7J). These effects were abrogated by EX527 co-treatment, consistent with the *in vitro* findings showing that adiponectin suppresses mTOR phosphorylation, downregulates Wnt/β-catenin pathways, activates autophagy, and negatively modulates SREBP-1 and its targets in a SIRT-1 dependent manner. To verify the *in vivo* effects of adiponectin on cellular lipid homeostasis, neutral lipid and free fatty acid contents were analyzed in tumor-derived breast cancer cells. The results showed that treatment with gAcrp led to a significant decrease in both cellular lipid reservation (Fig. 7K) and free fatty acid levels (Fig. 7L), which were prominently restored by EX527, clearly confirming the important role of SIRT-1 in adiponectin-promoted alterations in lipid metabolism.

## Discussion

Adiponectin, an adipokine predominantly derived from adipocytes, possesses prominent anti-breast cancer properties. It has been also reported to exert a wide range of metabolic actions underlying its protective role against metabolic disorders, such as type 2 diabetes, dyslipidemia, and non-alcoholic fatty liver disease [42, 43]. While its potent anti-cancer effects and various metabolic actions under physiological conditions have been well documented, little is known about the role of adiponectin in modulating cancer metabolism, a critical event in cancer cell survival and growth. In the present study, we have elucidated the effects of adiponectin on fatty acid metabolism in breast cancer cells, characterized by impaired cellular lipid pool resulting from decreased fatty acid synthesis and enhanced fatty acid degradation, which in turn crucially contributes to cancer cell fate decision. In addition, we provide the first evidence that SIRT-1 induction mediates metabolic actions and breast cancer cell death by adiponectin.

Fatty acids, other than the function as cellular fuel substrates, are building blocks of integral lipid species, including triglycerides, phospholipids, and sphingolipids, which can be further converted into more complex molecules in various metabolic pathways. Fatty acid derivatives are essentially required for the maintenance of membrane structure and fluidity, cellular energy homeostasis, second messenger-mediated signal transduction, and post-transcriptional protein modifications [44]. It is therefore unsurprising that many cancer cells show increased dependence on biosynthesis and/or exogenous uptake of fatty acids, offering an opportunity to counteract cancer progression. Based on the finding that adiponectin induces cellular lipid deficiency in breast cancer cells, we sought to determine the consequences of dysregulated lipid homeostasis in cancer cell life. Interestingly, we found that lipid depletion by adiponectin resulted in disorganization of lipid rafts, which are membrane microdomains critical for localization and functioning of numerous membrane proteins, including LRP6 of the wnt/β-catenin pathway and Akt of the PI3K pathway. It should be noted that although lipid rafts are cholesterol-rich microdomains, which are supposed to be especially sensitive to cholesterol deficiency, their integrity is also determined by cellular level and composition of fatty acids. Indeed, besides cholesterol, lipid rafts are enriched with saturated long-chain fatty acids and their derivatives [45]. Moreover, trafficking and cellular localization of raft-associated proteins have been proposed to be dominated by palmitoylation [46, 47], emphasizing the importance of palmitate and related molecules in the modulation of raft-dependent signaling pathways. In fact, a previous report as well as our present findings showed that FASN inhibitor TVB-3166 triggered a disruption in lipid raft architecture, leading to alterations in the localization of membrane proteins and interruption in signal transduction of various signaling pathways [48]. This notion was further confirmed by the finding that exogenous palmitate supplementation partly abolished apoptotic cell death by adiponectin and TVB-3166 (Fig. 2). Of relevance, while previous studies have speculated that suppression of β-catenin pathway and dephosphorylation of Akt are involved in the anti-breast cancer effects of adiponectin [49, 50], our study sheds light on a novel mechanistic model elucidating how adiponectin inhibits the signaling pathways involved in the survival of the breast cancer cells.

The cellular lipid pool is determined by various factors such as *de novo* lipogenesis, exogenous lipid uptake, and lipid consumption, among which elevated *de novo* FAS has been established as a common metabolic phenotype of many tumor types, including breast tumors [18]. It has been previously reported that SREBP-1, a key transcriptional regulator of FAS-related genes, is overexpressed in breast cancer tissues and may be considered a prognostic marker of breast cancer progression [12]. Likewise, FASN, a rate-limiting enzyme in the saturated long-chain fatty acid synthesis pathway, is an interesting target for breast cancer therapy [51]. Therefore, suppression of SREBP-1 and SREBP-1 modulated FAS genes by adiponectin may lead to a decrease in intracellular fatty acid availability and crucially contribute to breast tumor suppressive activity of adiponectin. Given that cancer cells can mobilize fatty acids from lipid droplets through lipolysis pathway mediated by autophagy/lipophagy and adipose triglyceride lipase (ATGL) activity [39, 52], lipolytic processes may also have a significant impact on cellular lipid homeostasis. In the present study, activation of lipophagy by adiponectin led to a short-term increase in intracellular levels of free fatty acids, which were subsequently converted into FAO for energy production (Fig. 4). Stimulation of lipophagy-mediated FAO caused a further imbalance in the production/degradation of fatty acids and long-term deficiency of cellular fatty acids as a final consequence. Intriguingly, previous evidence has suggested that adiponectin causes ATP depletion, which in turn leads to cytotoxic autophagy activation in breast cancer cells; however, the mechanisms by which autophagy activation triggers cell death has not been described in detail [4]. In light of our findings, it could be assumed that adiponectin-induced energy depletion stimulates autophagy/lipophagy to fuel FAO and partly compensates for inadequate ATP production. We and other research groups have previously demonstrated that the coupling between FAS and FAO facilitates cancer cell growth [17, 32, 53], whereas FAO induction accompanied by downregulation of FAS observed in this study promoted cell death, indicating a context-dependent role of FAO in the modulation of cancer cell fate. In addition to FAO and FAS, our preliminary findings also suggested that adiponectin decreases the expression of membrane lipid transporters, including CD36 and LDLR, which may further contribute to fatty acid depletion in breast cancer cells (Fig. S5). Although we have not estimated individual contributions of alterations in FAS, FAO, and lipid uptake, the present data support a multi-mechanistic model for decreased cellular lipid pool by adiponectin.

Considering the key role of SREBP-1 in the regulation of fatty acid metabolism, we have conducted many studies to elucidate the mechanism whereby adiponectin suppresses SREBP-1 activity. SREBP-1 is a transcriptional factor whose level and function are controlled by various transcriptional and post-transcriptional modifications [54]. The existing evidence suggests that p300-dependent acetylation is a critical post-translational modification that directly affects the stability and activity of SREBP-1 [40, 55]. As a result, deacetylases can modulate transcriptional activity of SREBP-1 through deacetylation and destabilization of its nuclear form. Herein, we demonstrated that SIRT-1induction plays a pivotal role in the post-translational modification and destabilization of SREBP-1 by adiponectin. We further found that SIRT-1 induction by adiponectin is mediated via ROS production-dependent mechanisms (Fig. S3A and S3B), although the detailed underlying mechanisms were not identified. Notably, the role of SIRT-1 in adiponectin-induced fatty acid metabolic remodeling may not be limited to SREBP-1 downregulation and suppression of FAS. Indeed, recent literature ha delineated that deacetylase activity of SIRT-1 is linked to the activation of lipophagy, partly by deacetylation of autophagy-related genes such as Atg5, Atg7, Atg8, and LAMP1 [52, 56]. In consistent with these observations, blockage of SIRT-1 resulted in abrogation of lipophagy activation by adiponectin (Fig. 5H), highlighting a multifaceted role of SIRT-1 in adiponectin modulation of fatty acid metabolism. This is the first report showing that SIRT-1 induction is essentially required for lipid metabolic remodeling of lipids and suppression of breast cancer growth by adiponectin. However, while SIRT-1 activation is linked to tumor promoter actions under certain conditions [57, 58], our findings support a context-dependent role of SIRT-1, which may be exploited to reverse tumor metabolic reprogramming.

Although the present study has mostly focused on the involvement of SIRT-1 in the metabolic actions of adiponectin, contributions of other signaling pathways could not be neglected. Compelling evidence shows that adiponectin modulates a wide range of signaling pathways involved in the survival and proliferation of cancer cells, many of which are critical modulators of cellular metabolism, such as AMPK and mTOR [3, 16]. Unsurprisingly, we found some evidence on a link between these pathways and altered fatty acid metabolism by adiponectin. For example, AMPK activation also contributed to SREBP-1 and FASN suppressive effects of adiponectin in breast cancer cells (Fig. S5), signifying that APMK is also implicated in the modulation of fatty acid metabolism by adiponectin. It is interesting to note the mutual interaction between AMPK and SIRT-1; for example, SIRT-1 deacetylates and activates LKB1, an upstream kinase in AMPK signaling pathway [59]. AMPK can also activate SIRT-1 by enhancing NAD^+^ level and/or Nampt activity [60]. In this study, we also observed that both AMPK and SIRT-1 signaling were required for the suppression of mTOR and FAS-regulated genes by adiponectin (Fig. S6 and Fig. 6, respectively), further supporting the notion that AMPK activation and SIRT-1 induction coordinate to reprogram fatty acid metabolism in adiponectin-treated breast cancer cells.

## Conclusion

**Fig. 8:**
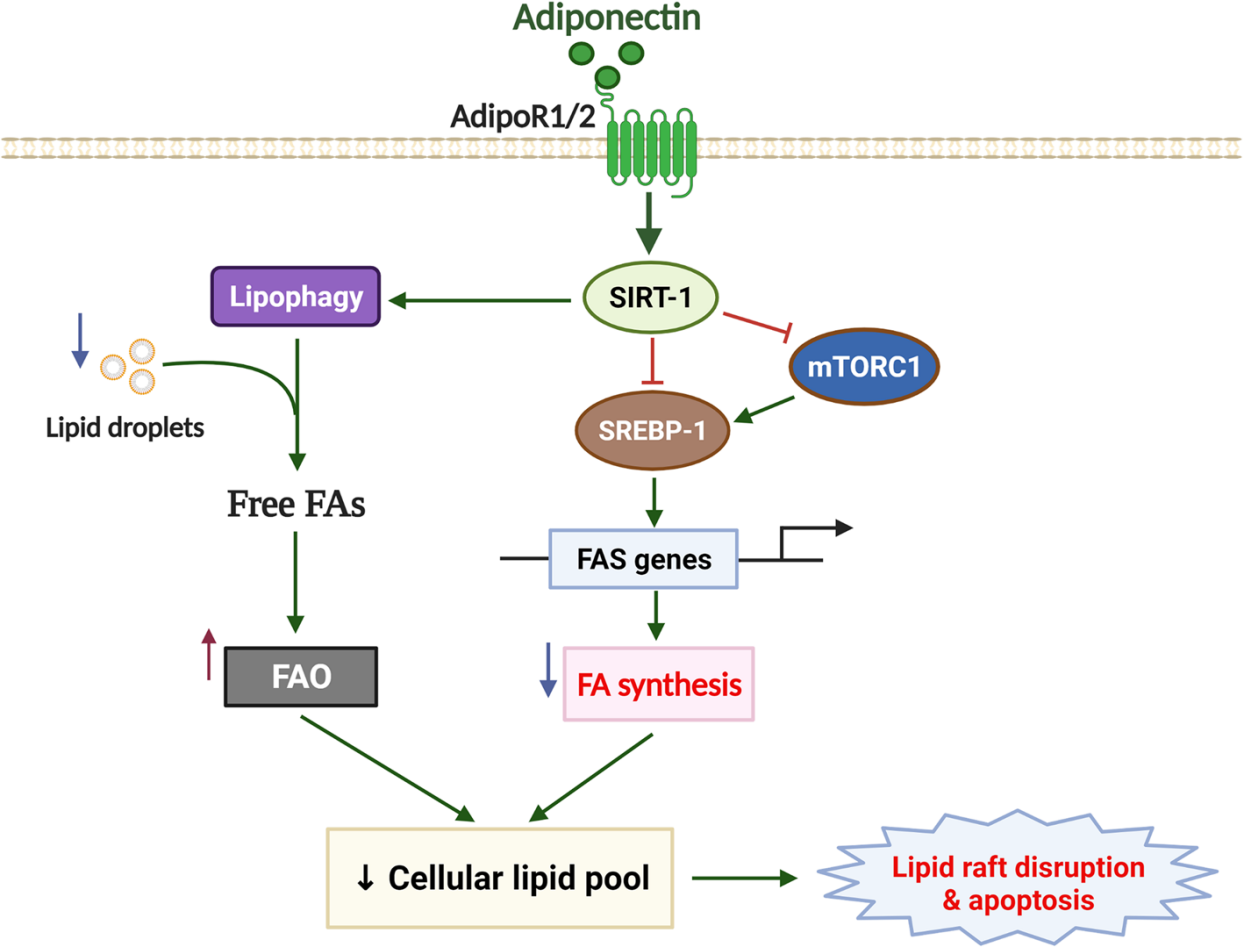
Proposed model for the modulation of tumor fatty acid metabolism by adiponectin. Adiponectin has been reported to potently suppress breast cancer growth. This study focuses on effects of adiponectin on tumor fatty acid metabolism and provides a novel mechanism for its breast cancer suppressing activity. SIRT-1 plays a central role in metabolic actions of adiponectin in breast cancer cells. On the one hand, SIRT-1 induction leads to downregulation of SREBP-1 by direct deacetylation and destabilization of nuclear SREBP-1 or by suppressing SREBP-1 expression through inhibition of mTOR signaling. SREBP-1 suppression leads to decreased expression of key enzymes in fatty acid synthesis (FAS) pathway and resultant blockage of FAS. On the other, SIRT-1 induction by adiponectin stimulates lipophagy to degrade lipid droplets and promote utilization of fatty acids for energy production via fatty acid oxidation (FAO). Inhibition of FAS accompanied by elevated FAO result in impairment in cellular fatty acid pool, which in turn causes disruption of lipid rafts and raft-dependent signal transduction, and cell apoptosis as a final consequence

In summary, the present study provides a novel insight into the molecular mechanisms underlying breast tumor suppression by adiponectin (Fig. 8). On the one hand, adiponectin suppresses lipogenesis by inhibiting the expression of SREBP-1 and its targets in the *de novo* FAS pathway. On the other, adiponectin also activates lipophagy, followed by degradation of fatty acids via FAO induction. Together, these metabolic alterations induced a deficiency in cellular fatty acid pool, which resultantly caused lipid raft disorganization, interruption of raft-associated signal transduction, and apoptosis in breast cancer cells. Finally, we highlighted the central role of SIRT-1 in fatty acid metabolic remodeling by demonstrating that SIRT-1 induction upon adiponectin treatment controlled the level of SREBP-1, either directly through deacetylation of its mature form or via modulation of mTOR phosphorylation, and activation of lipophagy. These findings suggest that activation of adiponectin signaling and its downstream effectors, such as SIRT-1, might be a promising strategy to counteract tumor-promoting metabolic reprogramming.

## Supplementary Information

**Additional file 1.**

**Additional file 2.**

**Additional file 3.** Sequences for siRNA and RT-PCR primers.

## Data Availability

All data generated or analysed during this study are included in this published article and its supplementary information files.

## Abbreviations

CT-B: Cholera Toxin Subunit B
FAS: Fatty acid synthesis
FFA: Free fatty acids
FAO: Fatty acid oxidation
gAcrp: Globular adiponectin
IHC: Immunohistochemistry
ROS: Reactive oxygen species
