# Supplementary figures and images for: Adiponectin triggers breast cancer cell death via fatty acid metabolic reprogramming
Source: J Exp Clin Cancer Res. 2022 Jan 5;41:9. doi: 10.1186/s13046-021-02223-y (PMC8729140; doi:10.1186/s13046-021-02223-y)

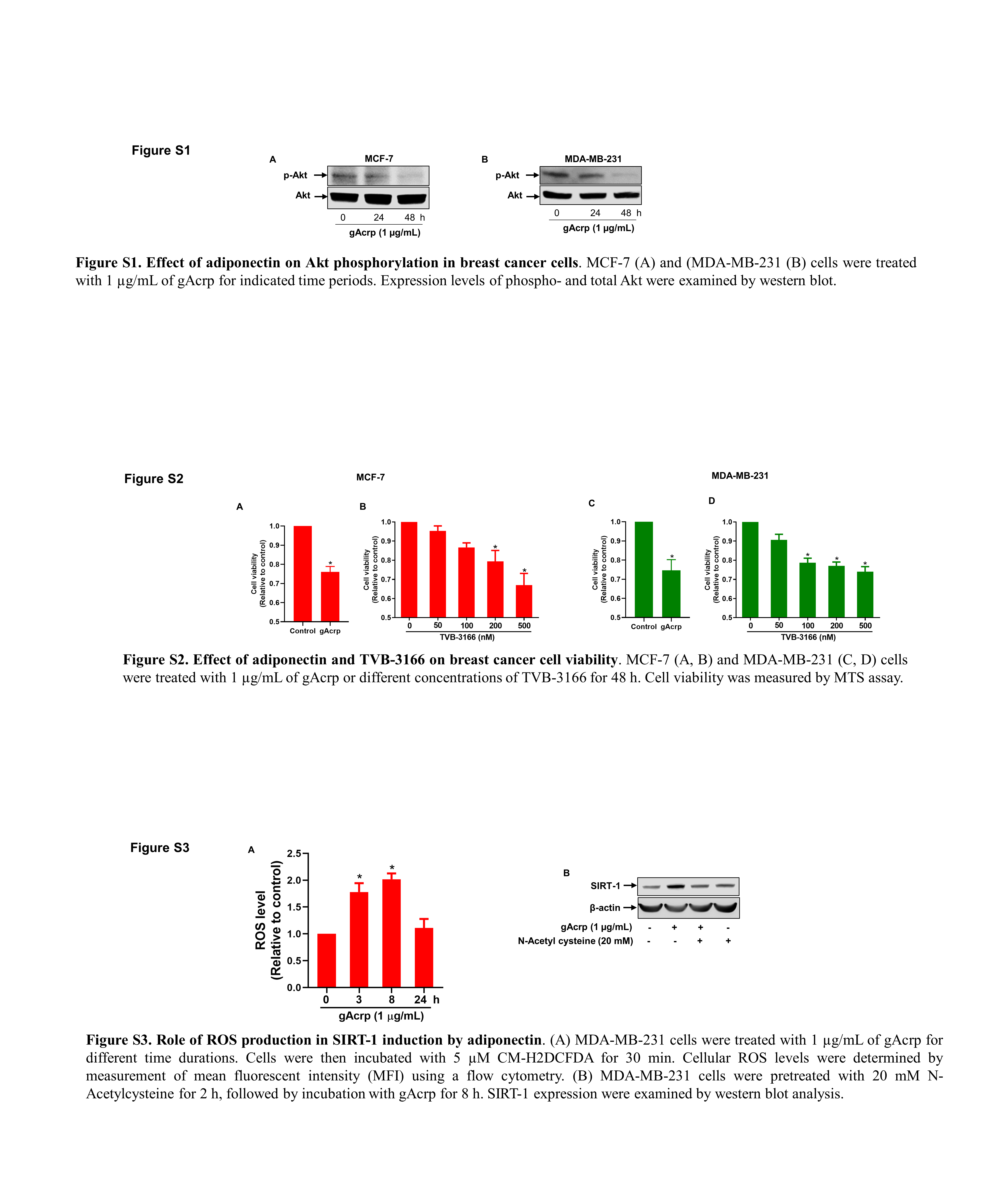

Supplement: Supplementary file 1 — Additional file 1. [file 13046_2021_2223_MOESM1_ESM.tif]

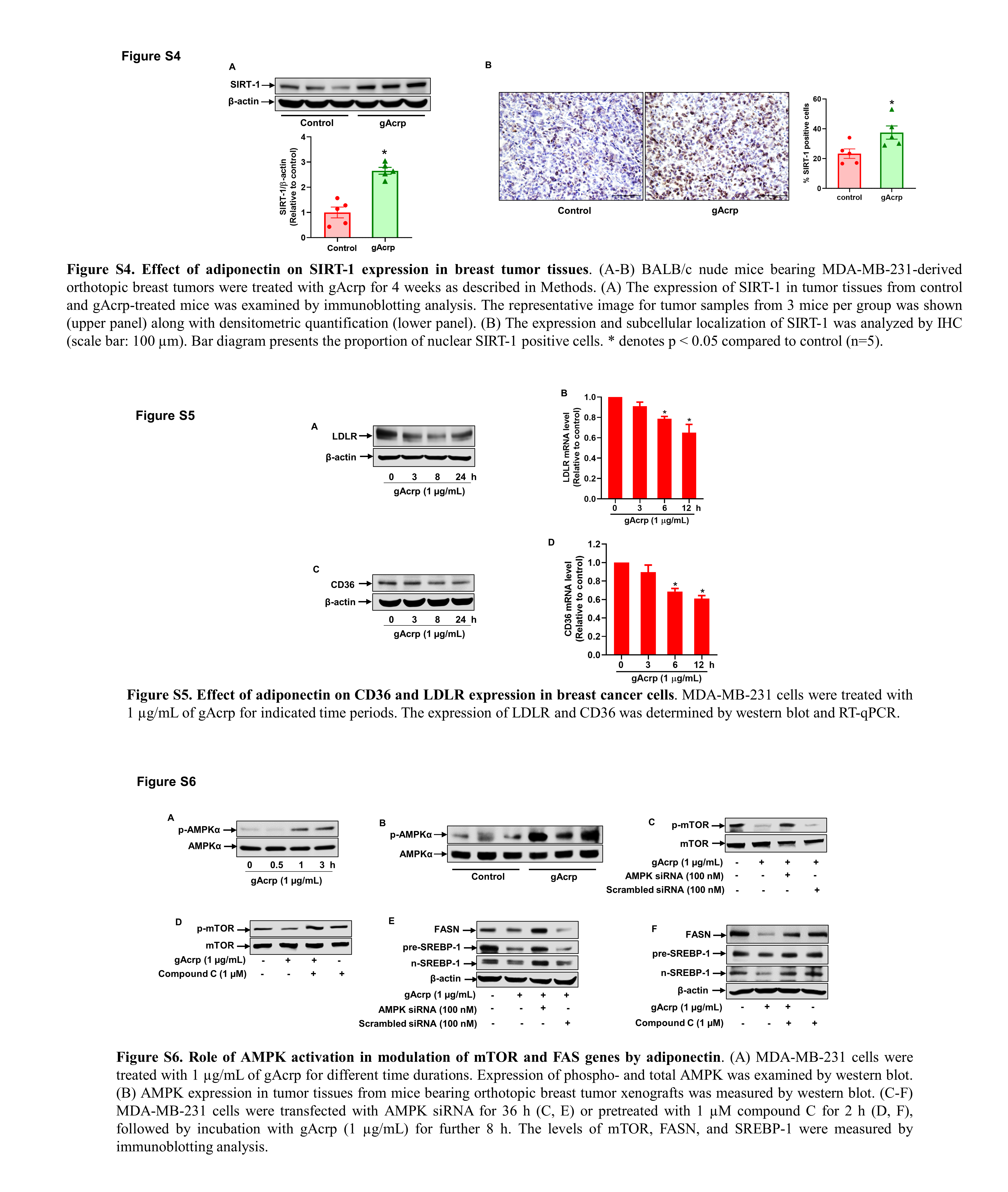

Supplement: Supplementary file 2 — Additional file 2. [file 13046_2021_2223_MOESM2_ESM.tif]
