# Supplementary material for: Adiponectin triggers breast cancer cell death via fatty acid metabolic reprogramming
Source: J Exp Clin Cancer Res. 2022 Jan 5;41:9. doi: 10.1186/s13046-021-02223-y (PMC8729140; doi:10.1186/s13046-021-02223-y)
Supplement: Supplementary file 3 — Additional file 3. Sequences for siRNA and RT-PCR primers. [file 13046_2021_2223_MOESM3_ESM.docx]

**Table S1.** Sequences for siRNA and RT-PCR primers

| **Target gene** | **Primer** | **Nucleotide sequence** |
| --- | --- | --- |
|  | **siRNA sequences** | |
| *SIRT-1* | F  R | 5'-GACUCUGAAGAUGACGUCU-3'  5'-AGACGUCAUCUUCAGAGUC-3' |
| *AMPK* | F  R | 5'-CUGAGUUGCAUAUACUGUA-3'  5'-UACAGUAUAUGCAACUCAG-3' |
| *Scramble control* | F  R | 5'-CCUACGCCACCAAUUUCGU-3'  5'-ACGAAAUUGGUGGCGUAGG-3' |
|  | **Primer sequences for RT-PCR** | |
| *GAPDH* | F  R | 5'-ACCACAGTCCATGCCATCAC-3'  5'-TCCACCACCCTGTTGCTGTA-3' |
| *ACC1* | F  R | 5'-ATGTCTGGCTTGCACCTAGTA-3'  5'-CCCCAAAGCGAGTAACAAATTCT-3' |
| *ACLY* | F  R | 5'-TGCAAAGTGAAGTGGGGTGA-3'  5'-TTTGGGGTTCAGCAAGGTCA-3' |
| *CD36* | F  R | 5'-CAGGTCAACCTATTGGTCAAGCC-3'  5'-GCCTTCTCATCACCAATGGTCC-3' |
| *FADS1* | F  R | 5'-ACAGTGACAAAAGGCTCGGA-3'  5'-ACTTGACCAGAGGCAGCTTT-3' |
| *FADS2* | F  R | 5'-TTGTGTGTGCGTGTTGTTGG-3'  5'-ACCAATCAGCAGGGGTTTCA-3' |
| *FASN* | F  R | 5'-AAGGACCTGTCTAGGTTTGATGC-3'  5'-TGGCTTCATAGGTGACTTCCA-3' |
| *LDLR* | F  R | 5'-GACGTGGCGTGAACATCTG-3'  5'-CTGGCAGGCAATGCTTTGG-3' |
| *SCD1* | F  R | 5'-AAAGCGAGGTGGCCATGTTA-3'  5'-TCATGCCTCAAAACTGCCCT-3' |
| *SREBF1* | F  R | 5'- GCAAGGCCATCGACTACATT -3'  5'- GGTCAGTGTGTCCTCCACCT -3' |
